# Patient factors and their impact on neutropenic events: a systematic review and meta-analysis

**DOI:** 10.1007/s00520-019-04773-6

**Published:** 2019-04-16

**Authors:** Pinkie Chambers, Yogini Jani, Li Wei, Emma Kipps, Martin D. Forster, Ian C. K. Wong

**Affiliations:** 1 UCLH-UCL Centre for Medicines Optimisation Research and Education, Pharmacy Department, University College London Hospitals NHS Foundation Trust, 235 Euston Road, London, NW1 2BU UK; 2 UCLH-UCL Centre for Medicines Optimisation Research and Education, UCL School of Pharmacy, 29-39, Brunswick Square, London, WC1N 1AX UK; 3 The Royal Marsden Hospital, Fulham Road, London, SW3 6JJ UK; 4 UCL Cancer Institute, Department of Oncology, University College London Hospitals NHS Foundation Trust, 72 Huntley Street, London, WC1 6DD UK; 5 Centre for Safe Medication Practice and Research, Department of Pharmacology and Pharmacy, University of Hong Kong, Hong Kong, China

**Keywords:** Neutropenia, Cancer, Chemotherapy, Neutropenic sepsis, Risk

## Abstract

**Background:**

Neutropenia is associated with an increased risk of mortality and hospitalisation. Strategies, including the prescribing of colony-stimulating growth factors (CSFs), are adopted when a high risk (> 20%) of neutropenic complications are seen in the clinical trial setting. With a diverse treatment population that may differ from the patient groups recruited to studies, appropriate prescribing decisions by clinicians are essential. At present, results are conflicting from studies evaluating the risks of certain patient attributes on neutropenic events; we aimed to aggregate these associations to guide future management.

**Design:**

A systematic review with a meta-analysis was conducted using the Preferred Reporting Items for Systematic Reviews and Meta-Analysis (PRISMA) statement. Studies were identified through a literature search using MEDLINE, EMBASE and Cumulative Index to Nursing and Allied Health Literature (CINAHL) databases from inception to December 1, 2017. Studies were included into a meta-analysis if they adjusted for confounders; analyses were conducted in STATA v 15.1 SE.

**Results:**

A total of 4415 articles were retrieved by the search with 37 meeting the inclusion criteria and 12 eligible for meta-analysis. Meta-analysis was conducted for increasing age and yielded a pooled odds ratio of 1.39 (1.11, 1.76, I^2^ = 24.1%), in our subgroup analysis of 4814 patients. Odds ratios for studies were pooled that reported associations for one co-morbidity compared to none and resulted in an overall odds of 1.54 (CI 1.09–2.09, I^2^ = 13.1%), including 9189 patients in total.

**Conclusions:**

Results can enhance current guidance in prescribing primary prophylaxis for treatments that either fall marginally under the internationally recognised 20% neutropenia risk.

**Electronic supplementary material:**

The online version of this article (10.1007/s00520-019-04773-6) contains supplementary material, which is available to authorized users.

## Background

Neutropenia is a well-recognised complication of chemotherapy, associated with an increased risk of infection, febrile neutropenia (FN), and as a consequence, can lead to mortality [1]. Interventions to prevent neutropenic events (NEs) such as FN can reduce incidence and associated complications. Inventions include chemotherapy dose reductions and delays, prescribing prophylactic antibiotics and more commonly prescribing primary prophylaxis with colony-stimulating factors (CSFs). This latter strategy is favoured to maintain dose intensity. Guidelines are available that recommend the use of CSFs when a risk of FN is 20% or greater [2, 3]. However, the start time and duration of treatment remain at the discretion of the patient’s clinician.

In clinical practice, decisions on the best strategy to prevent NEs in patients treated with chemotherapy are challenging. With a diverse treatment population varying in weight, ethnicity, age, and co-morbidity, judgement has to be made on appropriate treatments and management strategies. Toxicity information from clinical trials is used to guide whether CSF prophylaxis is indicated [4]. However, data on neutropenic complications from clinical studies may not be representative of the wider population and this has caused uncertainty in using toxicity information as a guide [5].

Internationally recognised guidelines reflect this doubt, advising that individual patient factors such as age, and line of treatment should additionally be considered alongside toxicity information to guide management decisions [2, 6]. Yet, inconsistencies exist in the reported studies for associations with factors such as increasing age and neutropenic events, where one study showed an increase in neutropenia risk and another reported a reduced risk [7, 8]. Additionally, there is no quantification of risk associated between factors and neutropenic events within guidelines, which is essential for clinical decision-making.

The aim of this review was to therefore investigate factors that have demonstrated influence on neutropenic episodes and synthesise their significance. There is already a recognition of the importance of the chemotherapy regimen and an additional understanding of other risk factors would enable clinicians to appropriately prescribe preventative measures.

## Methods

### Search strategy and selection criteria

This is a systematic review that includes a meta-analysis based on peer-reviewed academic articles. The Preferred Reporting Items for Systematic Reviews and Meta-Analysis (PRISMA) guidelines [9] were followed for reporting of the methods and findings. The review protocol was registered in the Prospero International Prospective Register of Systematic Reviews (CRD42018097263).

Studies were identified through a literature search, guided by the Population-Intervention-Comparison-Outcomes (PICOs) framework, using MEDLINE, EMBASE and Cumulative Index to Nursing and Allied Health Literature (CINAHL) databases, from inception to December 1, 2017. An example of the search strategy is given in supplementary material. Reference lists of articles were reviewed to identify additional relevant publications.

Articles were screened against the inclusion criteria in two phases, by author PC, titles and abstracts followed by full texts; a duplicate screen of 10% of articles was screened by a second researcher (ML). Any conflict or uncertainty was resolved through consensus agreement with author YJ.

Studies were included if they were published in English and included human subjects aged 18 and over that were receiving cancer chemotherapy. We included studies that were systematic reviews, randomised controlled trials or observational studies. The studies must have quantitatively evaluated the association between individual factors and any NE, i.e. FN, FN admission, dose delays due to neutropenia or laboratory-tested myelosuppression. Exclusions included early phase pharmacological studies, where the purpose was to evaluate a drug or drug effect. Additionally, book reviews, opinion articles, editorial reviews and articles published in only abstract form were excluded.

A data extraction form was developed and piloted independently by two of the researchers (blinded) using a random sample of five articles. The following were extracted by the two researchers for each article: study design, method of data collection, setting, population characteristics (tumour group), method of analysis, all risk factors investigated, outcomes measured and strengths of association reported for significant factors. Data were extracted for the adjusted odds ratios (OR), relative risk (RR) and hazard ratios (HR), 95% confidence intervals and *p* values, where reported.

Methodological quality of studies was assessed for bias. As there were no randomised controlled studies that met the eligibility criteria, we used a published, modified version the Newcastle-Ottawa tool to assess the quality of studies [10] that provided a more comprehensive understanding for study quality and any bias that may exist [11], including handling of missing data. Quality was rated as high, moderate or low, if the total scores were greater than 17, 12–16 and less than 12 out of the total 21, respectively. PC conducted all quality reviews with a second author (YJ), independently duplicating 5% of the reviews with agreement on all double reviewed articles.

### Statistical analysis

It is understood from previous studies that confounders can impact the strength of associations [12]. Hence, only studies which adjusted for confounders using appropriate statistical techniques were included in any meta-analysis. The pooled odds ratio was calculated for neutropenic events at different age groups and for one co-morbidity compared to zero, using random effects models. Due to the heterogeneity of the studies, it was not possible to aggregate other factors other than age and co-morbidity in the same way. The Q-test was performed to assess between-study heterogeneity, and calculated the I^2^ statistic, which expresses the percentage of the total observed variability due to study heterogeneity. A subgroup analysis was necessary to explore the variation of the effect of age on neutropenic events. In this subgroup, we only included articles that adjusted for the confounders, renal function and co-morbidity. All analyses were conducted in STATA v 15.1 SE.

## Results

### Identification of articles

**Fig. 1:**
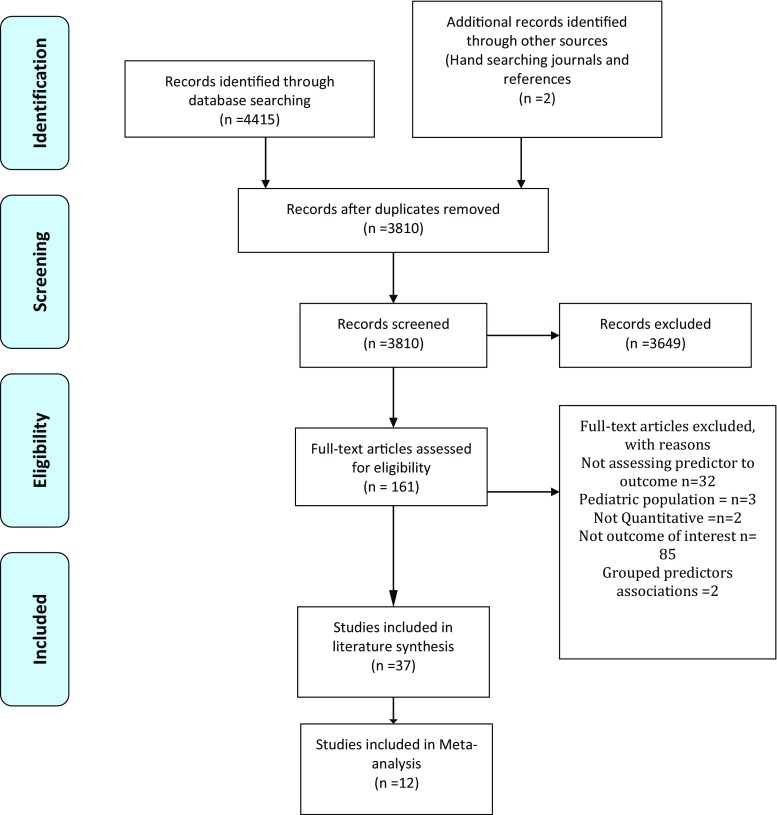
PRISMA flow diagram

The initial search returned 4415 published articles. Following title and abstract screening, 161 full-text articles were assessed against the inclusion and exclusion criteria, and 37 articles were included (Fig. 1). All identified articles were published between 2000 and 2017. The locations of these studies included the USA (*n* = 11) [7, 8, 13–21], Japan (*n* = 8) [22–29], the UK (*n* = 3) [30–32], Korea (*n* = 2) [33, 34], France (*n* = 1) [35], Canada (*n* = 3) [36–38], Belgium (*n* = 1) [39], India (*n* = 1) [40], China (*n* = 1) [41], and Spain (*n* = 1) [42]. Other studies involved multiple countries either through collection utilising collaboration [43–45] or utilising data available from randomised controlled studies [46, 47].

FN was the primary outcome measure for most studies [8, 13–20, 22, 24–26, 28, 30–40, 42, 45–48]. Other outcome measures used were grade 3 or above neutropenia [7, 15, 16, 19, 23, 24, 26, 27, 29, 35, 40–42, 44, 48], and dose delays and reductions [7, 24, 43].

Many studies included were in breast cancer, namely early breast cancer [20, 31, 33, 36–39, 43, 44] and a further 5 focussed on non-specific breast cancer [8, 13, 17, 21, 46]. Others investigated lung cancer patients [24, 27, 28, 30, 32, 41], patients with gynaecological malignancies [7, 14, 19], colorectal [25, 47], prostate [22, 26] and oesophageal cancers [29], myeloma [23], non-Hodgkin’s lymphoma [18, 34, 35, 45], and glioblastoma [40]. Other studies grouped 3 or more tumour types together [13, 15, 16, 42].

Overall, the factors identified were concordant with those found in a similar systematic review [49] and could be grouped into patient-, cancer- or treatment-related factors. Supplementary Table 2 outlines studies not included in the meta-analysis. Some authors aimed to develop predictive risk models, using findings from their research [16, 17, 25, 39, 45, 48]. These models would enable clinicians to score patient-related factors and calculate individual patient risk. Other authors focussed in detail into specific factors such as co-morbidity [15] or genetic influences [25] and articles only reported details of these associated hazard or odds ratios, despite recognising confounding factors were associated with the event. The 12 articles in Table 1 were included in our meta-analysis and the table describes details of confounders used in analysis.

**Table 1:** Studies included in meta-analysis (by year of publication)

First author, year and reference in brackets	Study design and country	Population (*n*), description of study sample	Outcome assessed	Significant patient factors, OR and confidence intervals	Factors assessed in multivariable model	Comments on quality
Fujiwara 2017 [28]	Retrospective study Observational Japan	*n* = 244 Lung	Febrile neutropenia	Male gender OR 4.26 (1.26–20.33) Radiotherapy pre-treatment. OR 6.09 (1.67–23.81)	Age, gender, ECOG PS, cancer type, stage, albumin, AST, total bilirubin, baseline neutrophil smoking, radiotherapy, surgery, chemotherapy treatment	High Authors failed to report missing data handling
Julius 2017 [7]	Retrospective study Observational US	*n* = 635 Gynaecological cancers	Chemotherapy-induced neutropenia including treatment delays and treatment dose reductions	Metabolic comorbidities** mainly diabetes mellitus Age-negative effect OR 0.865 (0.788–0.951)	Age, BMI, treatment, cancer type, stage, prior treatment (cycles received previously and regimen)	Moderate Authors failed to report missing data handling Population studied may influence generalisability
Naito 2017 [29]	Retrospective study Observational Japan	*n* = 66 Oesophageal cancer	Grade 3/4 neutropenia	Baseline platelet count OR 0.98 (0.97–0.99) ALT OR 1.15 (1.02–1.35) PPI administration OR 37.95 (3.53–1660.64)	Age, PPI treatment, baseline neutrophils and platelets. Albumin, ALT	Moderate Authors failed to report missing data handling Inadequately powered study.
Agiro 2016 [8]	Retrospective study Observational USA	*n* = 8745 Breast cancer TC *n* = 4815 TCH *n* = 2292 AC *n* = 1638	Neutropenic episodes Including febrile neutropenia	TC CSF OR 0.29 (0.22–0.39) age effect > 65 years (ref) 18–44 OR 0.52, 0.31 to 0.85 Comorbidity 0.65 0,42 to 1,00 CSF 0,29 0.22 to 0.39 TSH age effect > 65 years (ref) 18–44 OR 0.92 0.46 to 1.83 0.46 0.23 to 0.93 0.19 0.12 to 0.30 AC 1.21 0.75 to 1.93	Age, co-morbidity, stage, CSF use, prophylactic antibiotics	High Authors failed to report missing data handling
Kim 2016 [33]	Retrospective study Observational Korea	*n* = 610 Early breast cancer	Febrile neutropenia and relative dose intensity	GFR < 60 ml/min 2.806 (1.3–6.1) Age 2.804 (1.16–6.8) Co-morbidity 2.12 (1.078–4.536)	Age, co-morbidity, stage, renal function, WBC count, haemoglobin level, CSF use	High Authors failed to report missing data handling
Altwairgi 2013 [37]	Retrospective Observational Canada	*n* = 239 Early breast cancer	Febrile neutropenia	No patient-specific factors	Age, treatment, GCSF	High missing data not fully reported
Jiang 2013 [41]	Retrospective study Observational China	*n* = 141 Non-small cell lung	Grade 3–4 neutropenia	Age OR 3.819 (1.23–11.83) Albumin (g/dl) OR 3.3 (1.13–9.87) BSA > 2 OR 4.040 (1.45–11.22)	Age, weight, gender, PS, renal, diabetes, albumin, BSA	High missing data not fully reported
Lyman 2011 [16]	Prospective study Observational US	*n* = 3760 Cancers included colorectal (*n* = 521), small cell lung (*n* = 210), non-small cell lung (*n* = 697), ovarian (*n* = 312), breast (*n* = 1473), lymphoma (*n* = 547)	Neutropenic episodes FN or grade 3 or 4	Immunosuppressive medication OR 1.554 (1.105–2.187) AST > 35 u/L OR 1.422 (0.991–2.04) ALP > 120 u/L OR 1.469 (1.058–2.040) Bilirubin >1 mg/dL OR 2.152(1.235–3.747) Low baseline WBC OR 0.930 (0.8920–0.969) GFR OR 0.993 (0.989–0.997)	Age, prior chemotherapy, immunosuppressive medications, high AST, ALT or bilirubin, reduced white blood count or estimated GFR, patients with small-cell lung cancer, with planned RDI 85% CSFs	High All criteria met
Schwenkglenks 2010 [44]	Prospective study Observational Multinational	*n* = 444 Curative breast	Grade 4 neutropenia	Increasing age OR 1.35 (1.06–1.73) Increasing weight OR 3.85 (1.84–8.07) Vascular comorbidity OR 2.29 (1.25–4.20), baseline WBC OR 0.87 (0.76–0.99), higher baseline bilirubin OR 4.38 (1.25–15.33)	Age, weight, co-morbidity, liver, renal, FBC, RDI, CSF	High All criteria met in assessment
Pettengell 2008 [45]	Retrospective study Observational Belgium, France, Germany, Spain and the UK	*n* = 749 Non-Hodgkin’s lymphoma	Febrile neutropenia	Age OR 2.2 (1.21–4.01) Recent infection OR 3.07 (0.99–9.52) low baseline albumin < 35 g/l OR 4.76 (0.09–2.99)	Age, dose intensity, cycle 1 FN, CSF, renal, co-morbidity	High All criteria addressed
Dranitsaris 2008 [46]	Prospective study Observational Data from multicentre RCT	*n* = 509 Metastatic breast cancer	Any neutropenic complication > G2 NCI neutropenia or febrile neutropenia	Age > 59 OR 1.90 (0.95–3.78) PS WHO 2 OR 6.65 (2.54–17.39), cycle > 1 2.41 (1.32–4.39) Baseline neutrophil 2 × 10^9^ cells/L 4.25(0.99–18.2)	Age, treatment, PS, stage, number of cycles	Moderate Study did not fully address confounders. Missing data not reported
Schwenkglenks 2006 [43]	Retrospective study Observational Luxenberg, Belgium, France, Germany, Spain and the UK	*n* = 2860 Early breast cancer	Neutropenic events Delays or hospitalisations	Higher age 1.02 (1.01–1.03) Higher BSA 3.85 (1.84–8.07) Lower BMI, 0.003 Body mass index 0.97 (0.94–0.99)	Age, weight, treatment, diarrhoea, regimen, cycles, radiotherapy	High All criteria addressed

*ANC* absolute neutrophil count, *ALT* alkaline transaminase, *AST* aspartate transaminases, *BMI* body mass index, *BSA* body surface area, *CSF* colony-stimulating factors, *ECOG* Eastern Cooperative Oncology Group, *FBC* full blood count, *FN* febrile neutropenia, *GFR* glomerular filtration rate, *GCSF* granulocyte colony-stimulating factor, *N* numbers of patients, *PPI* proton pump inhibitor, *PS* performance status, *RCT* randomised controlled trials, *RDI* relative dose intensity, *UK* United Kingdom, *US* United States, *WBC* white blood cells

**Authors did not detail statistics of finding

Quality assessment identified that a number of studies included overall did not document their use of missing data [7, 8, 15, 19, 23, 27–29, 34, 37, 41, 42, 46, 50] and some failed to meet the minimum required sample size necessary to draw conclusions [23–27, 29]. In addition, when using univariable methods to choose factors to build into the multivariable models, some studies used a standard 95% significance level test [7, 8, 19, 23, 24, 26, 28, 29, 34, 37, 42, 50]. Although this is a recognised standard in many circumstances, in these types of studies, it is preferable to use a less rigorous cut-off to include factors that may become significant when adjusted for confounders. Despite this, for the majority, quality was high in all other domains of the assessment.

Of the patient-related factors, age was studied by 17 authors [7, 8, 16, 18, 19, 25, 26, 28, 29, 33, 34, 37, 41, 43, 44, 46, 51] with the majority finding older age to significantly increase NEs [8, 16, 18, 19, 25, 26, 41, 43–46, 50]. Conversely, one study involving 635 patients with gynaecological malignancies concluded that lower age increased the risk of NEs [7]. Additionally, in two other studies, age was found to be non-significant in univariable analysis [17, 39] and therefore was not investigated through multivariable methods. Pettengell et al. [45] found age to be significant in their risk model development study but actually noted that it could be interchanged with a marker of renal function. There was variation in the way that age was analysed: authors used either linear chronological age [25], or dichotomised age, such as using a threshold of greater than 65 years [8, 16, 18].

**Fig. 2:**
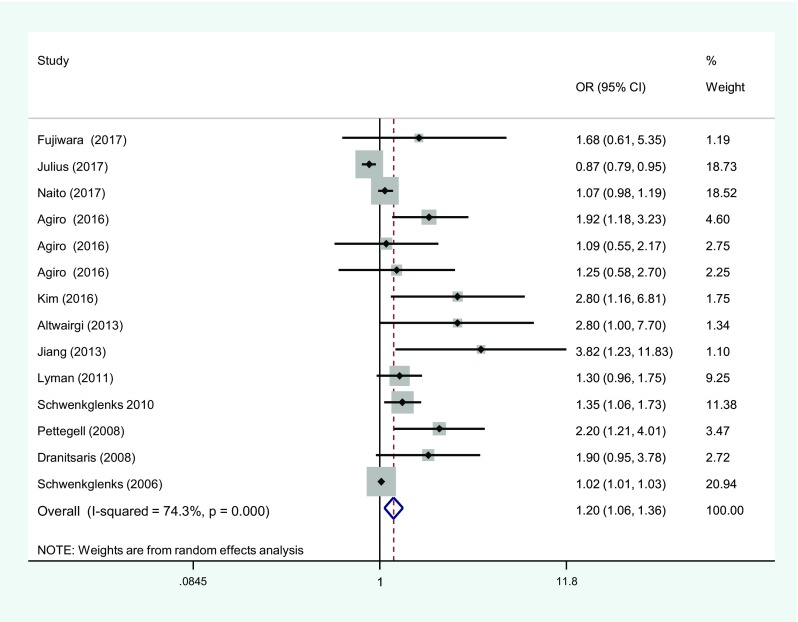
Meta-analysis showing pooled odds of neutropenic events with ages > 65 years. OR, odds ratios; CI, confidence intervals

**Fig. 3:**
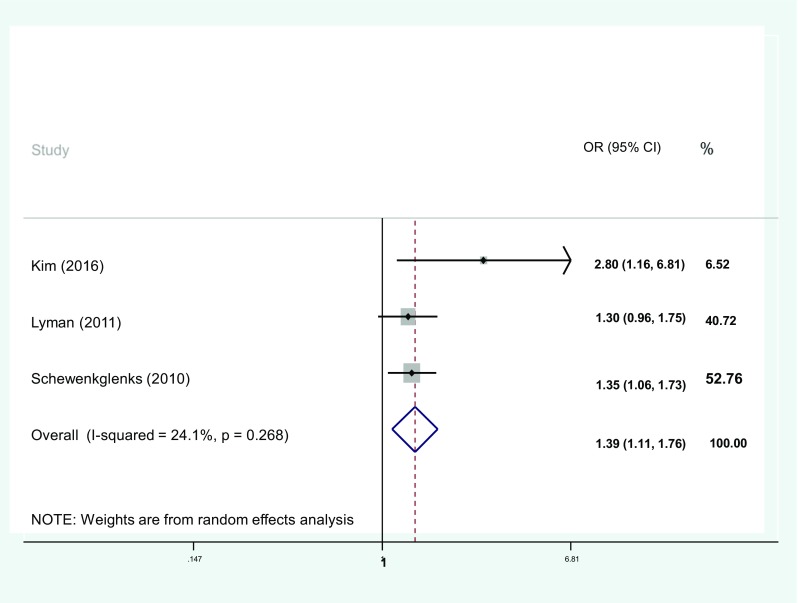
Subgroup analysis showing pooled odds of neutropenic events with > 65 years. Only studies adjusting for confounders included. OR, odds ratios; CI, confidence intervals

**Fig. 4:**
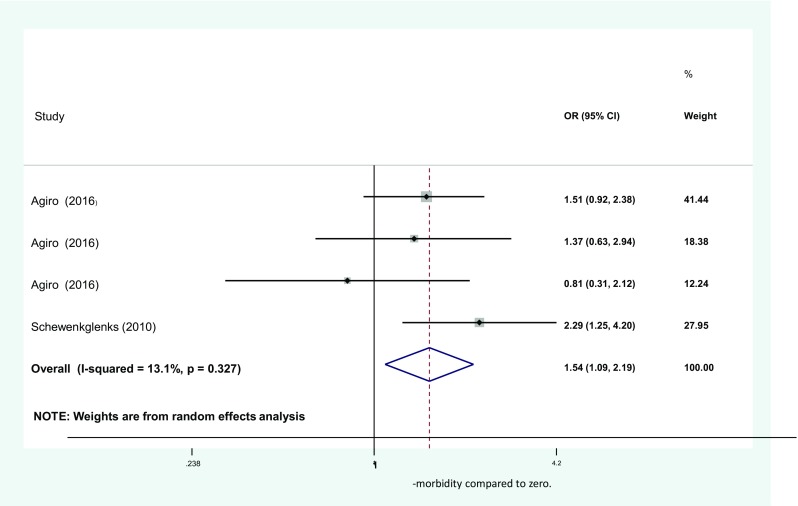
Meta-analysis showing pooled odds of neutropenic events with one co-morbidity compared to zero. OR, odds ratios; CI, confidence intervals

A meta-analysis of age where ORs were available and pooled yielded a combined OR of 1.2 (1.06–1.36) (Fig. 2). This included studies from a number of tumour groups, and in some cases, there was no adjustment for the confounders’ renal function and co-morbidity which may limit clinical acceptance. Additionally, when I^2^ was calculated, a high degree of heterogeneity was identified. A subanalysis that only included results from studies that adjusted for important confounders such as co-morbidity and either renal or liver function [16, 33, 44] (Fig. 3) yielded an OR of 1.39 (1.11, 1.76) (Fig. 4), with an acceptable level of heterogeneity (I^2^ = 24.1%). These 3 studies selected in the analysis included data from 4814 patients of which 2497 patients were treated for breast cancer.

A number of studies investigating co-morbidity [7, 8, 16–18, 26, 29, 33, 34, 41, 44, 45] found it to be a significant factor. However, we were only able to pool results from two articles due to the diverse methods in which co-morbidity was recorded. Within these, four independent studies were reported. Agiro et al. reported OR for patients receiving 3 separate chemotherapy regimens, where events and controls were independent for the different treatment groups. The aggregation of the four studies yielded an overall OR of 1.54 (CI 1.09–2.09) (see Fig. 4). This analysis included 9189 curative breast cancer patients. All studies in this meta-analysis included neutropenia-related hospitalisations encompassing FN in addition to treatment delays as measures of outcome. To add to this combined result, one very large study including 19,160 patients detailed the effect of individual co-morbidities and found that having three or more other conditions produced a HR of 1.73 (1.33–2.26) for FN. Conversely, a non-significant result was reported for grade 4 neutropenias [15], highlighting differences in mechanisms between NS and NEs.

Findings from smaller studies investigating markers of myelosuppression such as neutrophil, platelet and white cell counts or levels of haemoglobin prior to chemotherapy initiation [24, 25, 27, 29] were limited by inadequate sample sizes. Two larger studies [16, 44] did find that reduced white cell counts prior to treatment would increase the risk of NS; however, threshold values were unavailable from the articles. One study involving 509 breast cancer patients did conclude that a neutrophil value of < 2 × 10^9^/L at baseline would result in a 4.2-fold increase in the risk of any neutropenic complication [46]. Similarly, in a study investigating cycle 1 FN in 577 patients with non-Hodgkin’s lymphoma [18], a pre-treatment haemoglobin level of < 12 g/L resulted in a hazard ratio of 1.44 (1.03–2). Results could not be pooled due to differences in measurements and dichotomisation.

## Discussion

The aim of this review was to evaluate, via a meta-analysis, the associations between factors that have demonstrated influence on NEs, in order to guide future management of chemotherapy patients. A number of factors were frequently investigated and reported to be associated with NEs, including age, co-morbidity and baseline bone marrow suppression. We were able to aggregate the reported OR from included studies for both age and co-morbidity and determine the pooled effect. We found that increased age and the presence of just one co-morbidity increase the occurrence of NEs by approximately 40 and 50% respectively; these findings should be used to guide the management of patients.

Similar factors encountered in our study were also described in another systematic review conducted in 2014 [49]. However, in our review, by adding quantifications to factors related to NEs, we have enhanced understanding of the importance of personalised care. This is particularly relevant as we approach an era of pre-determined electronic prescribing protocols for chemotherapy and supportive care. The implications of our findings are more prominent in treatments that fall marginally short of the 20% neutropenic risk threshold that currently indicates use of CSFs. A combined OR of 1.39 for an age above 65 and 1.54 for one co-morbidity (compared with none) was found. In practice, the results could determine individualised CSF prescribing rather than simply using published toxicity data from clinical trials. In the cases of early breast cancer, where treatments such as docetaxel/cyclophosphamide in clinical trials have demonstrated a moderate risk of NEs of 15% [52], clinicians may want to consider primary prophylaxis.

Our findings cannot yet be fully incorporated into practice guidelines, primarily because there is yet to be a strong, prospective study that evaluates all factors that may affect NEs. These factors include those such as performance status, severity of co-morbidity and ethnicity. The recently updated NCCN guidance has acknowledged the increased risk with advanced age [53]; however, age could simply be a proxy measure for frailty [54] or organ function. Within the meta-analysis, only studies adjusting for confounders were included but frailty was not studied by any author, which limits our findings. The diversity in study methods and criteria for inclusion limited us to only being able to pool data for one co-morbid condition compared to zero. Equally, we could not aggregate results of baseline bone marrow function or the effect of renal and liver function tests. The influence of gender has also been recently highlighted as an area where further research is required [55] and should also be considered when assigning treatment. The majority of studies that were appropriate for meta-analysis included women receiving treatment for breast cancer. This limited evaluation of gender and may also limit the findings of the study to patients treated with breast cancer.

Despite only including studies that included similar confounders, we found heterogeneity was inherently present due to differences in collection methods. One source of heterogeneity was our grouping of grade 3–4 NE and FN as outcome measures. Interestingly, there were no reports of mortality in any of the articles, which may be an effect of the collection methods. Further work is needed to define how neutropenic episodes translate to mortality following the developments in rescue medications such as CSFs.

A prospective study incorporating the findings of our work could guide the development of a risk prediction model. We found a number of model development studies; however, many were excluded from our review as individual OR and HR were not reported. We have identified weaknesses with some of the risk prediction models included in our analysis, consistent with other models developed for use in cancer patients, limiting their current use.

Neutropenia is one of the most common and most dangerous AEs of chemotherapy. For this reason, a strategy to prevent the event occurring is essential. Trial data of new treatment regimens can help us to understand the effects of treatment on the bone marrow. However, these studies are often undertaken in a controlled group of patients and it is difficult to assess other patient-related factors that increase the risk.

## Conclusions

Our study has demonstrated that there are many patient-related factors that have influence on NEs. By determining the magnitude of risk of advanced age and co-morbidity, we have enhanced current guidance. However, further work is urgently needed in developing a comprehensive risk model to guide better patient management.

## Electronic supplementary material

ESM 1 (DOCX 24 kb)
