# Supplementary material for: Patient factors and their impact on neutropenic events: a systematic review and meta-analysis
Source: Support Care Cancer. 2019 Apr 16;27(7):2413–24. doi: 10.1007/s00520-019-04773-6 (PMC6541585; doi:10.1007/s00520-019-04773-6)
Supplement: Supplementary file 1 — (DOCX 24 kb) [file 520_2019_4773_MOESM1_ESM.docx]

**Supplementary Materials**

| *Cancer Chemotherapy identification* |
| --- |
| 1. exp Neoplasms/ |
| 1. cancer$ or neoplas$ or oncolog$ or malignan$ or tumo$ or sarcoma$ or leukaemi$ |
| 1. 1 or 2 |
| 1. exp Antineoplastic Agents/ |
| 1. exp Drug Therapy/ |
| 1. Antineoplastic Combined Chemotherapy Protocol* or Chemotherap* |
| 1. 4 or 5 or 6 or 7 |
| 1. 3 and 7 |
| *Risk factors* |
| 1. risk$ or risk factor$ or odds or caus$ or etiolog$ or predict$ |
| 1. Exp risk factors/ |
| 1. Exp causality/ |
| 1. Exp etiology |
| 1. 9 or 10 or 11 or 12 |
| *Myelosuppression* |
| 1. Leukopenia, this term only |
| 1. Exp Agranulocytosis/ |
| 1. Granulocytopen$ or agranulocyto$ or neutropen* or leu*open* or aplasia,aplastic,aplasion or nadir* |
| 1. 14 or 15 or 16 or 17 |
| *Final Search* |
| 8 and 13 and 17 |

*Supplementary material table : . Example based on OVID-Medline: was adapted for other databases*

| **Supplementary Table 2. Studies not included in meta-analysis (by year of publication)** | | | | |
| --- | --- | --- | --- | --- |
| **First author, year and reference (in brackets)** | **Study design, and country** | **Population (N), description of study sample** | **Outcome assessed** | **Patient predictors found significant** |
| Li 2016^13^ | Retrospective  Observational  US | N=3312  Multiple tumour groups | Febrile neutropenia | Only co-morbidity assessed : renal dysfunction, liver disease, and osteoarthritis |
| Mitani 2016^23^ | Retrospective study  Observational  Japan | N=47  Myeloma | Grade 3/4 neutropenia | Haemoglobin level |
| Ichikawa2015(75)^25^ | Retrospective Study  Observational  Risk model development  Japan | N=1312  Colorectal | Febrile Neutropenia  Severe grade 3-4 neutropenia. | Genetic factors  Baseline bilirubin  Neutrophils  Age |
| Ikesue 2015^24^ | Retrospective Study  Observational  Japan | N=77  Lung | grade 3-4 neutropenia or dose reduction or Febrile neutropenia | Haemoglobin level  Based on 1g/dl decrease  Neutrophils |
| Assi2014^36^ | Retrospective  Observational  Canada | N=251  Early breast cancer | Febrile neutropenia | None |
| Chao2014^15^ | Retrospective  Observational  US | 19 160, 963 with neutropenia  Multiple tumour groups | Febrile neutropenia, grade III and IV neutropenia combined, and grade IV neutropenia alone. | COPD, CHF, autoimmune disease,  peptic ulcer disease renal disease and thyroid disorder |
| Choi2014^34^ | Retrospective  Observational  Korea | 181  Diffuse large B Cell Lymphoma | Neutropenia and Febrile neutropenia | Female gender  Bone marrow involvement  Comorbid condition |
| Pfeil2014^39^ | Retrospective  Observational  Belgium | Early breast cancer  Total 994, of which 166 febrile neutropenia | Febrile neutropenia | Lower platelet count  haemoglobin at baseline  Patient height.  Genetics: certain polynucleotide polymorphisms,, |
| Shigeta2014^26^ | Prospective Study  Observational  Japan | N=95  Metastatic prostate cancer | Severe neutropenia, febrile neutropenia, | age ≥75 years number of comorbid conditions, history of radiotherapy |
| Shiota 2014^39^ | Prospective  Observational  Japan | N=37  Metastatic prostate cancer | Febrile neutropenia | Low serum albumin and low lymphocyte count |
| Gupta2013^40^ | Retrospective Study  Observational  India | N=107  Glioblastoma | Neutropenic episodes | Gender  BSA, BMI  Serum creatinine |
| Watanabe2013^27^ | Prospective Study  Observational  advanced lung treated with anthracycline  Japan | N=61  Advanced lung cancer treated with anthracycline | grade 3-4 neutropenia | lower haematocrit values  Female Gender |
| Jenkins2012^30^ | Retrospective  Observational  UK | N=263  Lung | Febrile neutropenia | Baseline neutrophils and leukocytes |
| Laskey2012^19^ | Retrospective Study  Observational  USA | N=326  Epithelial ovarian cancer | Febrile neutropenia | age>60  Caucasian race |
| Phippen2011^14^ | Retrospective  Observational  US | N=58  Gynaecological malignancies | Febrile neutropenia | Baseline haemoglobin and albumin. Score of patient generated global assessment |
| Shirdel 2011^38^ | Retrospective  Observational  Canada | N=35  Early Breast Cancer | Febrile neutropenia | Pre-cycle blood tests and day 8 |
| Hosmer 2010^17^ | Retrospective Study  Observational  US | N=86,693  Multiple Tumour groups | Febrile neutropenia | Increasing number of comorbid conditions 1 month from time of diagnosis to initiation of chemotherapy |
| Lopez-Pousa 2010^42^ | Retrospective Study  Observational  Spain | N=1194  Breast, trachea, colorectal, ovary and stomach | Febrile neutropenia or >grade 3 neutropenia. | Baseline lymphocyte and neutrophil counts(-ve effect)  ECOG performance status >2 |
| Jenkins 2008^31^ | Retrospective  Observational  UK | N=740  Early Breast | Febrile neutropenia, chemotherapy delay | Low baseline neutrophils, platelets or white cell counts. |
| Crawford2005^32^ | Prospective  Observational  UK | N= 239  Small Cell Lung Cancer | Febrile neutropenia | Gender |
| Hurria 2005^20^ | Retrospective  Observational  US | N=1405  Early breast cancer | Febrile neutropenia or grade 4 haematological toxicity. | Increasing creatinine |
| Meyerhardt2004^47^ | Retrospective  Observational  Multinational data from RCT | N=287  Colorectal | Febrile neutropenia | Bilirubin level |
| Lyman2003^18^ | Retrospective Study  Observational  US | N=577  Intermediate grade NHL | Neutropenic episodes | 1st FN associated with >65 age Cardiovascular disease  baseline Hb<12g/dl |
| Rivera2003^21^ | Prospective Study  Observational  US | N=143  Breast cancer | ANC < 0.5×109/litre neutropenia-related dose reduction of 15% or neutropenia-related dose delay of 7 days or more, or febrile neutropenia.. | Base line neutrophils |
| Voog2000^35^ | Prospective Study  Observational  France | N=101  Non-Hodgkin’s lymphoma | Grade 4 neutropenic event  Febrile neutropenia | **Performance status**  high levels of soluble p75-R-TNF |
| Abbreviations :RCT= Randomised Controlled Trials. US – United States. UK –United Kingdom N= numbers of patients | | | | |
